# Grazing Ecology of Sheep and Its Impact on Vegetation and Animal Health on Pastures Dominated by Common Ragwort (*Senecio jacobaea* L.)—Part 2: Animal Health

**DOI:** 10.3390/ani12101289

**Published:** 2022-05-18

**Authors:** Susanne Ohlsen, Martin Ganter, Peter Wohlsein, Bernd Reckels, Aiko Huckauf, Nikola Lenzewski, Sabine Aboling

**Affiliations:** 1 Institute of Animal Nutrition, University of Veterinary Medicine Hannover, Foundation, 30173 Hannover, Germany; bernd.reckels@tiho-hannover.de; 2 Clinic for Swine, Small Ruminants, Forensic Medicine and Ambulatory Service, University of Veterinary Medicine Hannover, Foundation, 30173 Hanover, Germany; martin.ganter@tiho-hannover.de; 3 Department of Pathology, University of Veterinary Medicine Hannover, Foundation, 30559 Hannover, Germany; peter.wohlsein@tiho-hannover.de; 4 Nature Conservation Foundation Schleswig-Holstein, 24113 Molfsee, Germany; aiko.huckauf@stiftungsland.de; 5 Institute of Plant Science and Microbiology, Universität Hamburg, 22609 Hamburg, Germany; nikola.lenzewski@uni-hamburg.de

**Keywords:** *Senecio jacobaea* L., sheep, ragwort intake, blood parameter, copper liver tissue values, animal health

## Abstract

**Simple Summary:**

Common ragwort (*Senecio jacobaea* L.) contains potentially toxic pyrrolizidine alkaloids (PA). Occurring on sites of roughage production, it contaminates the harvest. Regardless of fresh or dried intake, ragwort may result in fatal liver cirrhosis in livestock animals. Controlling ragwort, especially without biocides in nature conservation areas, is difficult. Since sheep seemed more resistant to PA in experiments, we tested how this animal species copes with ragwort under field and free-choice conditions. On a ragwort-rich pasture with a stocking density of 12 sheep/hectare, animals grazed for six months over two grazing seasons. From 70 sheep in the beginning, seven were slaughtered every six weeks for analysis of liver parameters, including seven control animals in the beginning of each grazing season. We addressed two questions: (1) To what extent do sheep voluntarily ingest ragwort on contaminated pastures? (2) Which impact on animal health does this grazing behavior have? Sheep ingested large amounts of ragwort, exceeding the assumed lethal dose established under experimental conditions by up to 200%. Behavior, body condition, and liver enzymes mainly remained unchanged. From the point of view of animal health and nature conservation, sheep grazing might be an option to reduce the amount of ragwort on pastures.

**Abstract:**

Common ragwort (*Senecio jacobaea* L.) naturally occurs on species-rich grasslands. Containing pyrrolizidine alkaloids (PA), it endangers livestock health through contaminated feed. Although in vitro studies showed a detoxification capacity of PA in sheep, few field data are available on the ability of grazing sheep to cope with ragwort. During two grazing seasons on a ragwort-rich pasture, we studied: (1) To what extent do sheep voluntarily ingest ragwort and (2) What impact their grazing behavior has on animal health. Ragwort intake was monitored by counting missing plant parts and calculating their weight. From 70 sheep, seven were slaughtered at the beginning and in six-week intervals at the end of each grazing period to monitor blood parameters and liver tissue. Sheep continuously preferred ragwort. The daily intake was above the currently assumed lethal dose, varying between 0.2–4.9 kg per sheep. Clinical, hematologic, and blood biochemistry parameters mostly remained within the reference limits. Initially elevated liver copper content declined over time. The liver of all 70 animals displayed slight to moderate hepatitis, fibrosis, and proliferation of the bile ducts, but no morphological signs of liver cirrhosis. Sheep preferred and tolerated ragwort, making their grazing an option to control ragwort from both an animal health and nature conservation perspective.

## 1. Introduction

Fresh ragwort (*Senecio jacobaea* L.) is known for its potential toxicity to livestock [1], especially horses and cattle in the case of scarce feed [2,3,4,5,6,7,8,9,10,11,12,13,14]. In addition, dried ragwort also keeps its toxicity, e.g., in hay [4,15,16,17] or when pressed into pellets or cubes, less so in silage [15,18,19,20]. The toxicity is due to pro toxic pyrrolizidine alkaloids (PA), which can be metabolized to toxic pyrroles in the liver and as such lead to deadly liver cirrhosis [1,15,21,22] or tumors [23,24,25]. Sheep, along with wild living ruminants [26], however, are an exception due to the high tolerance and detoxification ability in these animal species, [9,27,28] as well as high hepatic microsomal enzyme activity extensively catalyzing the hydrolysis of *Senecio* PA [29,30]. Moreover, their rumen biome is able to eliminate PA to a limited extent [9,28,31,32]. For several other animal species, ragwort feeding is known to be harmful and can even be fatal [10,11,32,33,34]. 

In Germany the contamination of grasslands with ragwort is an increasing phenomenon and often considered a problem [35]. For precautionary reasons the German Feedstuff Act [36] forbids the use of ragwort-contaminated vegetation as roughage. Apart from open questions on possible ecological damage, using animal ‘weed grazers’ on contaminated pastures would violate national regulations of the German Animal Welfare Law (TierSchG §3 Abs. 10) [37]. In the present study we addressed the risk of ragwort poisoning for grazing sheep and asked two questions: (1) To what extent do sheep voluntarily ingest ragwort on contaminated pastures? (2) Which impact does this grazing behavior have on animal health? 

## 2. Materials and Methods

### 2.1. Study Design

A total of 70 female sheep (White Polled Heath sheep and their cross breeds), consisting of 16 animals older than one year and 54 gimmers, participated in this study, which ran over two grazing seasons from May until October 2020 and 2021, respectively. The non-pigmented animals with white fleeces grazed on a 5.25 ha pasture in Northern Germany. The pasture was divided into nine pens of 0.58 hectare each, and in each pen nine 20 m² plots of 4.47 m × 4.47 m were randomly located and installed: six plots for monitoring the sheep’s browsing (54 browsing plots in total on the whole pasture) and three plots for botanical monitoring and sampling (27 botanical plots in total on the whole pasture). Hay from a hay rack was continuously provided ad libitum, as well as mineral lick for sheep. Additionally, minimal amounts of concentrates were offered to get the sheep to follow the investigators into a small corral for clinical investigations once a week. There was no stable or shelter on the pasture, but a row of trees on the pasture and adjacent hedgerows offered protection from the sun and inclement weather. Every two weeks we monitored the grazing impact by counting missing parts of ragwort (half and whole leaves as well as shoots left behind by the grazing sheep) and calculated their weight (original substance (OS)) by using reference material (half and whole leaves as well as shoots) collected on the pasture (cf. part one of this article [38]). Before the onset of grazing in the first year, seven control animals randomly selected by their ear tag numbers were slaughtered. In the further course of the study, seven more sheep were slaughtered every six weeks until the end of the first season. The remaining 35 sheep were removed from the study area at the end of October 2020 and kept on a ragwort-free winter pasture elsewhere. At the beginning of the second grazing season, seven sheep were slaughtered as a second control group and the others transported to the study area, where again seven animals were removed from the pasture and slaughtered every six weeks until the end of October 2021. Whenever a group of seven sheep was removed from the pasture, its size was reduced by 0.58 hectare to maintain a continuous stocking density of 12 sheep/hectare.

### 2.2. Supporting Animal Health

Before commencing with the study, the sheep received both a veterinary check-up and individual ear tags. They were vaccinated against bluetongue disease (Suvazyl BTV 4+8 (Laboratorios Syva, Leon, Spain)) and clostridiosis (Covexin 10 (Zoetis Deutschland GmbH, Berlin, Germany)), dewormed with 200 µg Moxidectin/kg body weight (Cydectin^®^ 0.1% oral Lösung; Zoetis Deutschland GmbH), and supplied with a rumen bolus containing iodine, cobalt, and selenium for juvenile animals (Smartrace lamb 24-7 (Argimin Ltd., Kirmington, UK)). Before the second grazing season in 2021, these treatments were repeated for the remaining 28 sheep, now using a rumen bolus for adult sheep (Rumin 180^®^, WdT eG, Garbsen, Germany).

### 2.3. Sampling of Blood and Liver Samples

Blood samples (EDTA, Heparin, and Serum monovettes; Sarstedt AG & Co. KG, Nümbrecht, Germany) were taken at slaughter. EDTA samples were used for hematologic investigations within 24 h. EDTA anticoagulated blood was used to analyze hemoglobin concentration and white and red blood cell count (hematology analyzer, Celltag alpha, Nihon Kohden Europe GmbH, Kleinmachnow, Germany). Packed cell volume was analyzed after centrifugation, and blood smear microscopy was performed for each sample. Erythrocyte indices were calculated. Heparin and serum samples were centrifuged (2000 g, 15 min, centrifuge Hermle Z326, HERMLE Labortechnik GmbH, Wehingen, Germany) and stored at 18 °C until analysis. After thawing clinical chemistry tests were run for each individual. Measurements were performed with routine methods [39]. Furthermore, liver samples from the right and quadrate lobes were taken after slaughter and analyzed histopathologically. The copper content of the liver tissue was also determined from these samples.

### 2.4. Parameters Measured

Apart from analyzing the copper content both in the vegetation (six samples in June 2020 and 2021) and in ragwort (two samples in June 2020 and 2021) via atomic absorption spectrometry, the health status of the animals was assessed using four parameters (Section 2.4.1, Section 2.4.2, Section 2.4.3 and Section 2.4.4). Furthermore, a histopathological analysis of the liver tissue was performed (Section 2.4.5). 

#### 2.4.1. Clinical Parameters 

Once a week sheep underwent a detailed veterinary check including skin, especially the ears, respiratory rate, conjunctival color, episcleral vessels, color of urine, and body condition score.

#### 2.4.2. Blood Parameters 

Analysis of trace elements was performed according to Helmer et al. (2021) [40]. Using aspartate aminotransferase (ASAT), liver enzymes were analyzed according to the optimized UV-test of the International Federation of Clinical Chemistry (IFCC). Glutamate-dehydrogenase (GLDH) was analyzed with the optimized standard method according to the recommendations of the Deutsche Gesellschaft für Klinische Chemie (German Society for Clinical Chemistry), and gamma-glutamyl transferase (GGT) was analyzed using the enzymatic color test of the IFCC.

#### 2.4.3. Copper Content of Liver Tissue 

Due to the direct impact of PA (or, to be more exact, their metabolites) on the liver, special interest was focused on liver-related parameters such as copper content and liver enzymes. For evaluating the copper content, two walnut-sized samples of liver tissue were taken from each sheep, one from the right lobe for histopathologic evaluation and one from the quadrate lobe. These samples also served to evaluate cobalt and selenium contents in the control groups. The copper content of the liver tissue was analyzed by atomic absorption spectrometry (Solaar M6, Thermo Fisher Scientific, Inc., Waltham, MA, USA). The liver tissue was broken down using a microwave extractor (StarT, MLS GmbH, Leutkirch im Allgäu, Germany).

#### 2.4.4. Number of Endoparasites 

Once per month a collective fecal sample was taken for a fecal egg count, applying a modified version of the combined sedimentation-flotation process of Benedek (1943) [41]. 

#### 2.4.5. Structure of Liver Tissue 

Diagnostically relevant cell types were screened for signs of, for example, anisokaryosis, hepatitis, fibrosis, as well as proliferation of the bile ducts.

### 2.5. Statistical Analysis

The statistical analysis was performed in R 4.1.2 (The R Foundation for Statistical Computing, Vienna, Austria). 

Groups were compared using paired-sample two tailed t-tests. Correlations were tested using Pearson’s coefficient. Findings were assumed to be significant at the level of *p* < 0.05. Data were related to six-week intervals, so-called grazing periods.

## 3. Results

### 3.1. Intake of Common Ragwort

In the first grazing period of 2020, the sheep ingested, according to our counting, 10,393 half leaves of common ragwort (3.77 kg OS), 11,311 complete leaves (8.37 kg OS), and 4510 shoots (57.2 kg OS) on an area of 1080 m^2^ (54 browsing plots à 20 m^2^, cf. Section 2.1). Extrapolated to the pasture area, the whole flock ingested 3368.73 kg OS common ragwort during these first six weeks of grazing. This corresponded to 53.5 kg OS per sheep within the first grazing period and 1.19 kg OS per sheep and day. Surprisingly, in the first grazing period of the second year, the 28 remaining sheep ingested almost the same total number of half (*n* = 9991) and whole leaves (*n* = 15,852) and thus the same amount of plant material (15.4 kg OS) as the complete flock of 63 sheep at the beginning of 2020 (*n* = 10,393 (half leaves) and *n* = 11,311 (whole leaves) equals 12.14 kg OS). 

The copper content of the whole vegetation varied between 10.7 and 15.3 mg/kg DM (2020) and 14.0 and 16.0 mg/kg DM (2021). In ragwort we measured 16.8–18.4 mg/kg DM in 2020 and 17.7–20.2 mg/kg DM in 2021. Compared to a mean of 12 mg/kg DM reported in [42] for extensive pasture vegetation, our findings are slightly higher in the case of the vegetation and clearly higher in the case of ragwort.

### 3.2. Impact on Animal Health

#### 3.2.1. Clinical Symptoms

**Figure 1:**
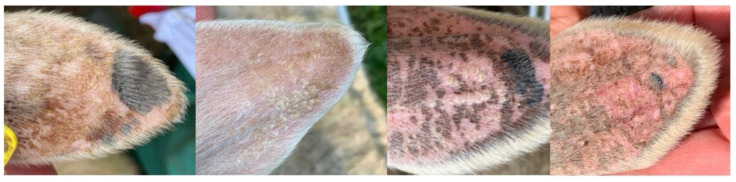
Small distortions of sunburn, pigment changes, crusts, and blisters at the ears.

None of the pastured sheep displayed any clinical symptoms that could be attributed to possible ragwort poisoning. Their body condition score ranged between 3.5 and 4 at the end of the study period. Apart from some cases of minor to moderate signs of lesions (Figure 1), which all healed spontaneously, diseases such as photosensitization (that has been reported in white animals grazing on ragwort-infected pastures [43,44]), icterus, or weight loss (usually accompanied by plant poisoning [45]) were absent. 

One sheep was found dead on the pasture on October 20th in the first year. The post-mortem report stated pyogranulomatous pneumonia caused by *Streptococcus ovis* as well as a liver abscess.

#### 3.2.2. Blood Parameters

**Figure 2:**
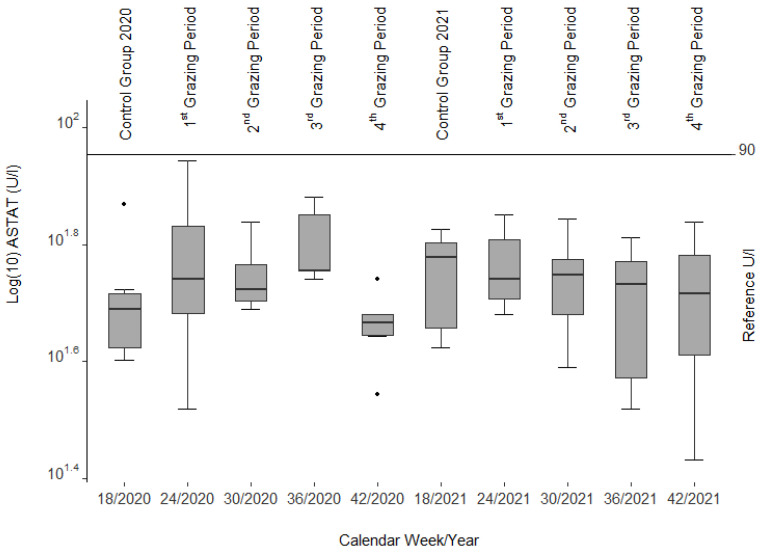
Aspartate aminotransferase (ASAT) activities from blood serum of the slaughtered sheep. Note the three single statistical outliers.

The ASAT activities were clearly within the reference range (30–90 U/L [39], cited in [40]). Except for the sudden decline in this enzyme during the 4th grazing period at the end of the first year (Figure 2), there were no significant changes. 

**Figure 3:**
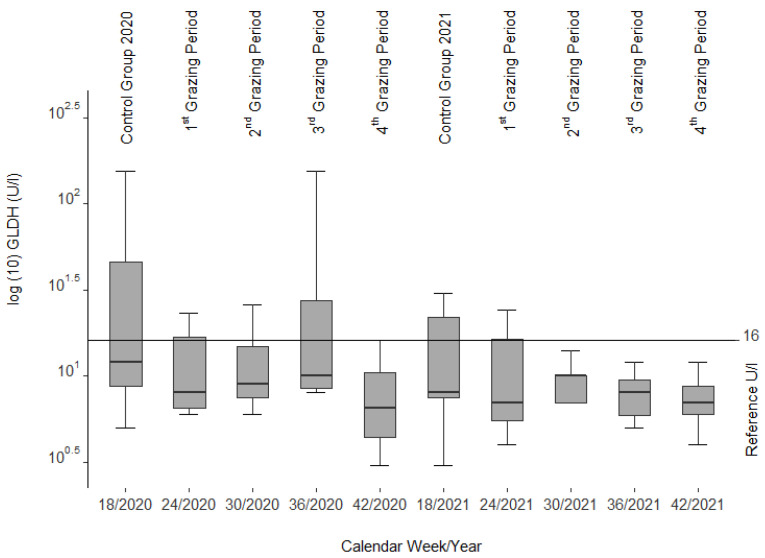
Glutamate dehydrogenase (GLDH) activities from blood serum of the slaughtered sheep.

The median GLDH activities (Figure 3) remained with slight variations (1st and 2nd grazing period in 2020 and control group and 1st grazing period of 2021) and two clearly higher variations (control group and 3rd grazing period of 2020) within the reference range (1–16 U/L [39], cited in [40]) as well. 

**Figure 4:**
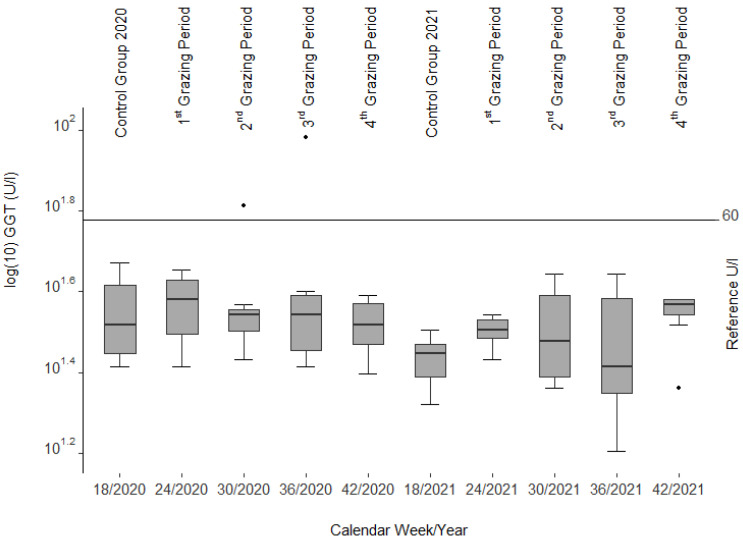
Gamma-glutamyl transferase (GGT) activities from blood serum of the slaughtered sheep. Note the three single statistical outliers.

The GGT activities (Figure 4) showed only little variance and were mostly within or only slightly outside the reference range of adult sheep (24–60 U/l [39], cited in [40]). We had three cases of minor hemoconcentration (packed cell volume (PCV) > 0.44 L per liter (l/l)) and one animal with a minor case of anemia (PCV < 0.22 l/l) along with leukocytosis (leucocytes 13.7 giga per liter (G/l)).

Clinical as well as hematological parameters (differential cell count, PCV, hemoglobin, erythrocyte indices, and bilirubin indices (see supplementary Table S1) did not show any significant changes over the course of time. 

#### 3.2.3. Copper Content of Liver Tissue

**Figure 5:**
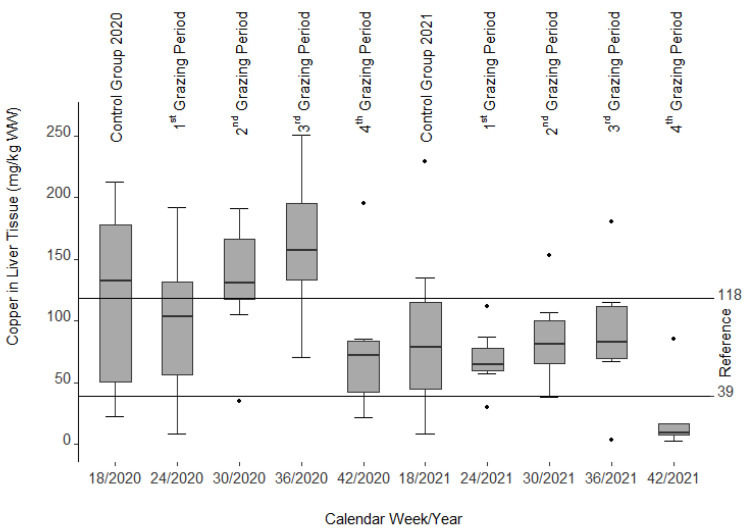
Copper content of fresh liver tissue (wet weight (WW)) after slaughter. Note the nine single statistical outliers.

Except for the 3rd and 4th grazing period in 2020, median values of copper content in the liver were within the reference range (39–118 mg/kg wet weight (WW)) [46,47] (Figure 5). In both grazing seasons (2020 and 2021), the copper content increased from the 1st to the 3rd grazing period and dropped thereafter. During the second grazing season (2021), liver copper concentrations were overall lower than in the first year, but also increased moderately (not significantly) from the 1st to the 3rd grazing period. In total there was a strong variation with values between 2.4 and 250.7 mg/kg WW. However, the same was true for the first control group, where values ranged from 22.0 to 212.5 mg/kg WW. Moreover, there was a significant decline in the liver copper content from a median of 117.6 mg/kg WW in the first control group in 2020 to a median of 77.1 mg/kg WW in the pasture grazing animals at the end of the first year (*p* = 0.015). A similar decline was also observed in the second grazing season with a median value of 90.7 mg/kg WW at the beginning of 2021 compared to 20.9 mg/kg WW at the end of 2021 (*p* = 0.048). Compared to the 4th grazing period in 2021, all copper values showed a significant decline (*p* = 0.003–0.048).

#### 3.2.4. Histologic Examination of the Liver Tissue

A total of 81.4% of all animals (*n* = 57 out of 70 sheep), including both control groups with seven animals each, displayed focal or multifocal slight to moderate lymphohistiocytic hepatitis. In contrast, the number of eosinophilic granulocytes surprisingly decreased during the second year. This diagnosis affected 85.7% of the animals in the first control group (*n* = 6) and 57.1% in the second one (*n* = 4). Likewise, focal or multifocal mild proliferation of the bile ducts was often observed in the control animals in both years (71.4% (*n* = 5) in 2020 and 57.1% (*n* = 4) in 2021). About 22% (*n* = 15 out of all 70 sheep) also showed focal or multifocal slight to moderate fibrosis. Apart from mild anisokaryosis of hepatocytes, the liver tissue of all animals was regularly structured with no signs of liver cirrhosis. 

The first control group in 2020 already showed minor hepatitis, fibrosis, and bile duct proliferations. As described above, this might be an incidental finding that could be normal, especially in gimmers.

#### 3.2.5. Endoparasites

Routine fecal egg count revealed only a very mild infestation with gastrointestinal nematodes during the whole study. Eggs of flukes and larvae of lung worms were not detected. Further deworming during the grazing season was not necessary in both years. 

## 4. Discussion

In this study we wanted to know to what extent sheep ingest ragwort on contaminated pastures under free-choice conditions and the impact the voluntary intake has on animal health. As to the impact on health, we differentiated between four variables (1. clinical symptoms, 2. blood parameters, 3. copper content of liver tissue, and 4. number of endoparasites). Additionally, the liver tissue (WW) was histopathologically evaluated.

### 4.1. Extent of Voluntarily Ingested Ragwort 

One of the most important findings of our study is that the observed ingestion of ragwort exceeds any lethal dose that was found under artificial conditions in feeding experiments [10,48] as well as in field experiments [34] or under conditions of restricted grazing [49]. The lethal doses of common ragwort may differ largely from >2–4 kg fresh common ragwort/kg body weight (>120–240 kg fresh ragwort/60 kg sheep) [17,50] (secondary sources) up to 200–300% of body weight [51] (>120–180 kg fresh ragwort/60 kg sheep). At the end of the first grazing season, each sheep had ingested an average of 360 kg of fresh common ragwort, exceeding the lethal dose (2–4 kg fresh ragwort/kg body weight) for an adult 60-kg sheep by 50–200%. 

The daily fresh ragwort intake of 4.9 kg/sheep in the second grazing period in 2020 might appear high. However, it is within the capacity range of possible feed intake after converting OS into DM. In 2020 the DM content of ragwort, averaged over all four grazing periods, was approximately 21% due to the forming of stalks, whereas in 2021 the average was clearly reduced to approximately 14%. The above-mentioned intake of 4.9 kg fresh ragwort per sheep in the second grazing period of 2020 corresponds to 1.03 kg DM. Given the age of the sheep (mainly 1–1.5 years), their daily feed intake varied between 0.8 and 1.4 kg of DM per day/sheep [52,53], proving sheep are capable of metabolizing the significant amounts of fresh ragwort. 

Two theses regarding PA metabolism in sheep are currently discussed: toxin breakdown in the digestive tract [54] or in the liver [54,55] as well as a sufficient serum albumin concentration [56]. Our field study supports the experimental finding (Craig et al. 1992a and Wachenheim et al. 1992b, cited in [56]) that ovine ruminal fluid containing 3.0 × 10^7^ PA-degrading bacteria/mL degraded PA within 2–6 h (as compared to bovines with 1.1 × 10^7^ bacteria/mL and a degradation time of 24–48 h). The free-choice grazing in our study proves the fundamental finding that sheep can cope with poisonous plants if they are not forced to feed on them [57]. Furthermore, the importance of a sufficient albumin concentration in serum is postulated in the context of chronic PA toxicity as hypalbuminemia might be followed by edema [56] and ascites [54]. 

### 4.2. Impact of Grazing Behavior on Animal Health 

#### 4.2.1. Clinical Symptoms

None of the sheep showed any clear symptom of PA poisoning. According to the pathology report, the only lethal case in a flock of 63 animals was most likely due to a pyogranulomatous pneumonia caused by *Streptococcus ovis*. Furthermore, no typical symptoms of PA intoxication such as veno-occlusive disease (VOD) of the liver, recently referred to as sinusoidal obstruction syndrome (SOS) in humans [58], which occludes small branches of the hepatic veins, causes ascites, edema, and reduced urinary output and can lead to cirrhosis and death [56], were noticed during our study. Instead, the sheep were continuously in good health and body shape. Towards the end of the second grazing season, their body condition scores exceeded 3.5, proving that the animals were in excellent shape. As all animals were slaughtered in the course of the study, statements regarding fertility were not possible.

#### 4.2.2. Blood Parameters

Hematologic parameters did not show any significant changes that could be seen in direct connection with the exposure to common ragwort over the period of two grazing seasons. Even though serum enzymes might not be perfectly suitable for diagnostic purposes in this context as their elevation may be transient and only occurs with actual tissue necrosis [54], the fact remains that the enzyme activities of the study sheep were mainly within the reference range.

In most cases, serum liver enzyme activities increase in livestock before recognizable liver lesions become clinically visible [32], cited in [56] as noted in calves and horses fed *Senecio jacobaea* [54]. Among those are glutamate dehydrogenase (GLDH), aspartate aminotransferase (ASAT), and gamma-glutamyl transferase (GGT). No such findings could be confirmed in our study as these values remained within the reference limits during both grazing seasons. However, a decrease in ASAT and GLDH (see Figure 2 and Figure 3) was correlated with the drop in liver copper content, a phenomenon that cannot be explained. 

GLDH activities of the control group in the first year varied. This is probably related to the grazing location where the animals were born and kept until the start of the study; however, we have no evidence to support this thesis. The cases of hemoconcentration are probably due to the transport to the slaughterhouse and/or the slaughtering process. The individual with minor anemia was an older sheep, and the cause of this slight anemia could not be identified.

#### 4.2.3. Copper Content of Liver Tissue

PA poisoning can cause excessive storage of copper, often resulting in fatal hemolytic disease [58,59]. However, our findings do not confirm this phenomenon for three reasons.

First, copper accumulation is increased in sheep with subclinical intoxication, even if the total copper intake declines due to the activation of copper-binding enzymes [60]. Therefore, copper accumulation might only be increased by PA consumption if the sheep are already in a subclinical stage of copper intoxication. Unfortunately, the liver copper concentrations of the control group slaughtered initially before the sheep went to pasture showed the widest variation in comparison with the animals slaughtered later in the study. Since the control sheep did not show homogeneous copper contents of the liver, it would have been difficult to identify any PA-related effect of copper accumulation. 

Second, the copper level in the liver increases due to the impairment of normal subcellular excretory mechanisms, including defects in lysosomal elimination of cell waste (Cheeke 1991, cited in [56]). Such a development could not be observed as the copper levels decreased significantly from the first control group in 2020 to the last group (4th grazing period) in 2020 and from the second control group in 2021 to the last group (4th grazing period) in 2021. 

Third, our data did not show the phenomenon of increasing copper tissue values after the termination of copper intake [60]. Instead, copper concentrations in the liver tissue remained on a plateau. In fact, about 45 days after the ragwort intake had decreased in the 4th grazing period of 2021, they dropped so much that the animals even showed a copper deficiency. Overall, despite all expectations, no cumulative increase in copper in the liver could be determined during this two-year project. Instead, the copper content of the liver tissue seems to reflect the amount of ragwort ingested by the sheep over both years (cf. part one of this article [38]), shifted by 45 days, as mentioned above. In the second year the amount of both present and ingested ragwort biomass almost halved compared to the first year. This is also reflected by the figures of copper content in the liver.

#### 4.2.4. Structure of Liver Tissue

Histopathologic findings associated with PA poisoning are mainly described for cattle or horse liver samples, where the PA intoxication results in focal hepatocyte necrosis, minimal peribiliary fibrosis, and mild bile duct proliferation. With time, damaged hepatocytes often develop into large megalocytes, resulting later in necrosis with subsequent inflammation, fibrosis, and ultimately cirrhosis [61]. In the pastured sheep of the present study, there was no obvious correlation between the time of exposure to common ragwort and the histopathologic findings. The cause of the observed inflammatory and reactive changes remained undetermined. On the other hand, 25% of the sheep showed a focal or multifocal mild to moderate liver fibrosis. Therefore, this extent of liver cell damage might be normal. 

#### 4.2.5. Number of Endoparasites

Surprisingly, no deworming was necessary during both grazing seasons. The reason for this could be phenols, which are part of the genus *Senecio* [62] and known to have anthelmintic effects [63]. Less deworming would be a welcome side effect not only for the animal’s health but also for the environment, where the administration of anthelminthic drugs poses a severe threat, e.g., for coprophagous beetles [64].

## 5. Conclusions

To our knowledge this is the first study proving that sheep are—under free-choice conditions—able to ingest significantly larger amounts of common ragwort than the assumed lethal doses established under experimental conditions [35,58,65]. We could not confirm any of the well-known detrimental effects of ragwort poisoning. In the course of our study, none of our four health parameters (clinical symptoms, blood parameters, copper content of the liver, and number of endoparasites) showed any conspicuous change, as well as the structure of the liver. On the contrary, we saw well-nourished and healthy animals in need of neither acute medical treatment nor deworming. 

## Data Availability

Data are contained within the article.

## Supplementary Materials

The following supporting information can be downloaded at: https://www.mdpi.com/article/10.3390/ani12101289/s1, Table S1: Blood Parameters; Median (Minimum, Maximum) in 2020 and 2021.
