# Supplementary material for: Grazing Ecology of Sheep and Its Impact on Vegetation and Animal Health on Pastures Dominated by Common Ragwort (Senecio jacobaea L.)—Part 2: Animal Health
Source: Animals (Basel). 2022 May 18;12(10):1289. doi: 10.3390/ani12101289 (PMC9137555; doi:10.3390/ani12101289)
Supplement: Supplementary file 1 [file animals-12-01289-s001.zip › animals-1658867-supplementary.pdf]

Blood Parameters; Median (Minimum, Maximum) in 2020 and 2021

		Group 1	Group 2	Group 3	Group 4	Group 5	Group 6	Group 7	Group 8	Group 9	Group 10
**Blood Parameters**	**Reference Values**	**Control Group 2020**	**1st Grazing Period 2020**	**2nd Grazing Period 2020**	**3rd Grazing Period 2020**	**4th Grazing Period 2020**	**Control Group 2021**	**1st Grazing Period 2021**	**2nd Grazing Period 2021**	**3rd Grazing Period 2021**	**4th Grazing Period 2021**
**Leukocytes**	(2.7-13.0 G/l)	8.5(5.5-13.9)	7.7(4.7-17.7)	7.5(3.8-9.4)	6.7(4.9-8.0)	6.7(5.7-13.7)	7.4(4.4-12.4)	7.4(5.3-10.0)	6.3(3.4-10.4)	7.5(4.3-8.7)	6.8(5.7-9.0)
**Erythrocytes**	(8.7-12.9 T/l)	12.7(10.9-14.7)	13.0(11.1-13.8)	12.6(11.5-14.9)	10.8(9.2-13.6)	9.2(5.5-10.9)	11.6(6.9-13.5)	11.0(10.3-12.6)	10.9(8.6-13.2)	9.6(5.5-11.0)	11.0(9.4-12.6)
**Hemoglobin**	(85-133 g/l)	118.5(110-135)	130(119-140)	125(111-146)	114(100-134)	108(87-126)	127(84-143)	123(114-145)	122(111-137)	114(87-123)	120(113-132)
**Hematokrit**	(0.25-0.41 l/l)	0.4(0.4-0.4)	0.4(0.4-9.4)	0.4(0.3-0.5)	0.4(0.3-0.4)	0.3(0.2-0.4)	0.4(0.3-0.4)	0.4(0.4-0.4)	0.4(0.3-0.4)	0.4(0.3-0.4)	0.4(0.4-0.4)
**MCV**	(34-46 fl)	30.1(28.0-33.0)	30.6(27.7-35.2)	30.9(28.3-31.9)	31.5(29.4-41.4)	37.5(30.3-41.3)	33.6(31.9-37.8)	34.9(32.5-36.4)	34.7(30.0-40.6)	35.6(32.4-50.9)	33.0(31.7-39.4)
**Monocytes**	(%)	1.3(0.0-4.5)	0.5(0.0-2.0)	1.5(0.5-2.5)	1.0(0.5-2.0)	0.8(0.0-1.5)	0.5(0.0-2.0)	0.0(0.0-1.0)	1.5(0.0-2.0)	0.5(0.0-1.5)	0.0(0.0-2.5)
**Lymphocytes**	(2.2-9.2 G/l)	4.0(2.0-7.8)	5.4(2.4-15.5)	4.5(2.5-5.0)	3.0(2.2-3.3)	3.7(2.4-6.8)	3.3(1.9-4.2)	2.9(2.5-5.1)	2.7(1.9-5.7)	3.9(3.2-4.3)	3.7(2.7-6.7)
**Segm. Granulocytes**	(0.1 - 5.0 G/l)	3.1(2.0-11.5)	2.0(0.7-4.8)	2.6(1.2-4.2)	3.0(1.6-4.3)	2.2(1.8-5.4)	3.7(2.2-8.3)	2.7(1.6-4.3)	3.2(0.7-4.0)	2.7(0.3-4.2)	2.8(1.0-3.7)
**Rod-nucl. Granulocyes**	(0-0.2 G/l)	0.0(0.0-0.1)	0.0(0.0-0.0)	0.0(0.0-0.0)	0.0(0.0-0.2)	0.0(0.0-0.0)	0.0(0.0-0.1)	0.0(0.0-0.0)	0.0(0.0-0.1)	0.0(0.0-0.0)	0.0(0.0-0.0)
**Eos. Granulocytes**	0-2.0 G/l	0.2(0.0-0.4)	0.2(0.0-0.3)	0.2(0.1-0.6)	0.2(0.0-0.5)	0.6(0.2-1.5)	0.3(0.0-2.0)	1.1(0.4-1.8)	0.6(0.2-0.8)	0.3(0.0-1.1)	0.3(0.1-1.3)
**Bas. Granulocyes**	(0-0.5 G/l)	0.0(0.0-0.1)	0.0(0.0-0.1)	0.1(0.0-0.2)	0.0(0.0-0.0)	0.0(0.0-0.2)	0.0(0.0-0.1)	0.0(0.0-0.2)	0.1(0.0-0.1)	0.1(0.0-0.1)	0.0(0.0-0.2)
**Monocytes**	(0-2.0 G/l)	0.1(0.0-0.5)	0.1(0.0-0.2)	0.1(0.0-0.2)	0.1(0.0-0.2)	0.1(0.0-0.1)	0.1(0.0-0.1)	0.1(0.0-0.0)	0.1(0.0-0.1)	0.0(0.0-0.1)	0.0(0.0-0.2)
**Copper in Liver Tissue (WW)**	(39-118 mg/kg/WW)	132.0(22.0-212.5)	103.5(8.1-191.6)	130.9(35.1-191.2)	157.3(70.6-250.7)	72.0(21.2-195.0)	78.7(8.1-229.3)	64.9(29.8-111.8)	81.2(37.7-152.8)	82.7(3.6-180.0)	9.5(2.4-85.0)
**Bilirubin**	(1-10 micromol/l)	1.6(0.3-4.5)	1.3(0.5-3.7)	4.6(3.2-6.5)	4.2(2.7-6.2)	2.9(0.5-4.2)	2.9(0.8-10.1)	2.2(1.2-3.3)	4.1(3.3-5.4)	3.0(1.2-4.1)	2.5(1.9-3.3)
**Protein**	(51-73 g/l)	73.6(60.3-79.7)	71.3(60.6-83.3)	71.6(66.9-84.3)	75.2(64.2-83.9)	77.5(71.7-82.7)	74.3(66.0-77.6)	77.9(70.7-84.3)	80.7(71.6-85.4)	81.8(76.4-87.1)	80.9(76.6-83.4)
**Albumin**	(35-38 g/l)	34.9(24.8-38.8)	31.9(28.1-35.3)	33.6(30.8-36.7)	34.0(30.2-39.0)	32.6(29.5-35.4)	31.8(29.1-35.3)	33.0(27.6-37.3)	34.0(31.9-35.1)	34.0(32.1-36.2)	34.6(31.2-37.7)
**ASAT**	(30-80 U/l)	49(40-74)	55(33-88)	53(49-69)	57(55-76)	46.5(35-55)	60(42-67)	55(48-71)	56(39-70)	54(33-54)	52(27-69)
**GLDH**	(1-16 U/l)	12(5-154)	8(6-23)	9(6-26)	10(8-155)	6.5(3-16)	8(3-30)	7(4-24)	9.8(6.6-13.6)	7.5(5.2-11.7)	7(4-12)
**GGT**	(24-60 U/l)	33(26-47)	38(26-45)	35(27-65)	35(26-96)	33(25-39)	28(21-32)	32(27-35)	30(23-44)	26(16-44)	37(23-28)
